# Hyperleukocytosis: A report of five cases and review of the literature

**DOI:** 10.3892/ol.2014.2326

**Published:** 2014-07-08

**Authors:** JUNMEI GONG, BIJIA WU, TIANJIAN GUO, SILANG ZHOU, BENFU HE, XINZHAO PENG

**Affiliations:** Department of Haematology, Chinese People’s Liberation Army No. 421 Hospital, Guangzhou, Guangdong 510310, P.R. China

**Keywords:** hyperleukocytosis, leukostasis

## Abstract

Hyperleukocytosis (white blood cell count, >100×10^9^/l), an uncommon presentation of leukemia, is associated with an increased risk of early mortality. It may present with a variety of symptoms secondary to leukostasis, a syndrome caused by the sludging of circulating leukemic blasts in the microvasculature. Adequate measures for managing this medical emergency include hydration, cytoreduction, prevention of tumor lysis and, rarely, leukapheresis in cases complicated by leukostasis and hyperviscosity syndrome. The present study reports a case series of five patients with hyperleukocytic leukemia. In addition, a review of the literature with regard to the incidence, pathophysiology, clinical manifestations and management of this laboratory abnormality is included. This study demonstrated that the central nervous system and lungs are the most common sites for leukostasis, and that emergency cases require aggressive treatment.

## Introduction

In patients with acute lymphocytic leukemia (ALL) or acute myeloid leukemia (AML), the clinical presentation is usually non-specific, including fatigue, fever or bleeding. However, certain patients with acute leukemia present with clinical signs of hyperleukocytosis (1). Hyperleukocytosis, defined as a white blood cell (WBC) count of >100×10^9^/l (100,000/μl), is often associated with increased morbidity and mortality in patients with leukostasis. Leukostasis is rapidly progressive and fatal in the majority of patients (2,3). The current study presents five patients with hyperleukocytosis. Cases 1, 2 and 3 were AML with induced leukostasis syndrome, while cases 4 and 5 were ALL without leukostasis syndrome. Patients provided written informed consent.

## Case reports

### Case 1

In March 2013, a 66-year-old female was admitted to the Department of Gastroenterology, Chinese People’s Liberation Army No. 421 Hospital (Guangzhou, China) following two months of decreased appetite and weight loss. Initial laboratory tests revealed a WBC count of 121.79×10^9^/l, hemoglobin count of 71 g/l (normal range, 100–160 g/l) and platelet count of 95×10^9^/l (normal range, 100–300×10^9^/l). The patient was transferred to the Department of Haematology due to suspected acute leukemia. On physical examination, the patient had a temperature of 39.4°C (normal range, 36–37°C), respiratory rate of 36 breaths per minute (normal range, 16–18 breaths per minute), heart rate of 132 beats per minute (normal range, 60–90 beats per minute) and blood pressure of 110/70 mmHg (normal range, 90–140/60–90 mmHg). A chest computed tomography (CT) scan showed a diffuse high-density mass shadow in the two lungs. Secondary laboratory tests revealed a WBC count of 144.23×10^9^/l, hemoglobin count of 65 g/l and platelet count of 48×10^9^/l. Bone marrow smear and immunophenotyping studies showed AML [acute monocytic leukemia subtype (M5)], according to the French-American-British classification on the basis of morphological features, and histochemical staining of cells with peroxidase and esterase (4). Fluorescence *in situ* hybridization of the bone marrow obtained from bone marrow puncture showed myeloid/lymphoid (or mixed-lineage) leukemia gene-negative. Blood gas analysis revealed the following: pH, 7.481 (normal range, 7.35–7.45); partial pressure of oxygen, 51.7 mmHg (normal range, 80–100 mmHg); partial pressure of carbon dioxide, 32.8 mmHg (normal range, 35–35 mmHg); bicarbonate ion concentration, 23.9 mmol/l (normal level, 24 mmol/l); and base excess concentration, 0.5 mmol/l (normal level, 0±2.3 mmol/l). Hydroxyurea (1.0 g three times a day) and cytarabine (100 mg twice a day) were administered by oral and intravenous infusion, respectively. Three days following transferral to the Department of Haematology, the patient succumbed to the disease.

### Case 2

In September 2012, a 50-year-old female was admitted to the Department of Haematology, Chinese People’s Liberation Army No. 421 Hospital following one month of recurrent oral ulcers and weight loss. Initial laboratory studies revealed a WBC count of 80.28×10^9^/l, hemoglobin count of 120 g/l and platelet count of 95×10^9^/l. Bone marrow smear revealed AML (M5). Three days following admittance, the patient experienced left arm numbness and developed coma during the night, and the WBC count increased to 282×10^9^/l. An emergency head CT scan showed cerebral infarction. The patient’s family abandoned all treatment and discharged the patient from hospital. The patient succumbed to the disease 5 h later.

### Case 3

In March 2012, a 51-year-old female was admitted to the Department of Haematology, Chinese People’s Liberation Army No. 421 Hospital due to a cough lasting for five days, expectoration and two days of gum bleeding. The patient’s WBC, hemoglobin and platelet counts were 41.73×10^9^/l, 9.9 g/dl and 28×10^9^/l, respectively. Additionally, the patient’s alanine aminotransferase level was 157.3 U/l (normal range, 8–40 U/l) and the aspartate aminotransferase level was 103.7 U/l (normal range, 5–34 U/l), while blood bilirubin, urea nitrogen and creatinine levels were normal. Bone marrow smear showed AML (M5). On day three, the patient experienced a severe headache and left arm numbness, with projectile vomiting, Laboratory tests revealed a WBC count of 131.82×10^9^/l, hemoglobin count of 10 g/dl and platelet count of 24×10^9^/l. An emergency head CT scan showed cerebral hemorrhage. The patient’s family abandoned all treatment and discharged the patient from hospital. The patient succumbed to the disease 8 h later.

### Case 4

In January 2009, a 23-year-old male was admitted to the Department of Haematology, Chinese People’s Liberation Army No. 421 Hospital due to multiple abdominal distensions in the past month and low-grade fever for the past two days. On physical examination, the patient demonstrated a fever (temperature, 37.4°C), and spleen and left cervical lymph node enlargement were appreciable. Sternal tenderness was also evident. Laboratory tests revealed a white WBC count of 253×10^9^/l, hemoglobin count of 110 g/l and platelet count of 120×10^9^/l. Bone marrow smear showed ALL (L2), and immunophenotyping studies showed acute B-lymphocytic leukemia. Fluorescence *in situ* hybridization of the bone marrow obtained from bone marrow puncture showed breakpoint cluster region (BCR)/Abelson (ABL) fusion gene-positive. Dexamethasone (15 mg per day) was administered by intravenous infusion for five days, to induce leukoreduction. This was then followed by combination chemotherapy, including vincristine (2 mg intravenous injection on days one, eight, 15 and 22), daunorubicin (40 mg/m^2^ intravenous infusion on days one to three, and days 15 and 16), cyclophosphamide (mesna rescue; 750 mg/m^2^ intravenous infusion one days one and 15), L-asparaginase (6,000 IU/m^2^ intravenous infusion on days 11, 14, 17, 20, 23 and 26) and predisone (1 mg/kg/day orally on days one to 14, with a decrease to one-third of the dosage on days 15–28). The patient acquired complete response after 58 days.

### Case 5

In May 2013, a 43-year-old female was admitted to the Department of Haematology, Chinese People’s Liberation Army No. 421 Hospital due to multiple headaches in the past month. Laboratory studies revealed a WBC count of 125×10^9^/l, hemoglobin count of 60 g/l and platelet count of 55×10^9^/l. Bone marrow smear showed ALL, and immunophenotyping studies showed acute B-lymphocytic leukemia. Fluorescence *in situ* hybridization of the bone marrow obtained from bone marrow puncture showed BCR/ABL fusion gene-positive. Dexamethasone (15 mg per day) was administered by intravenous infusion for five days, to induce leukoreduction. Subsequently, the patient received combination chemotherapy, including vincristine (2 mg intravenous injection on days one, eight, 15 and 22), daunorubicin (40 mg/m^2^ intravenous infusion on days one to three, and days 15 and 16), cyclophosphamide (mesna rescue; 750 mg/m^2^ intravenous infusion one days one and 15), L-asparaginase (6,000 IU/m^2^ intravenous infusion on days 11, 14, 17, 20, 23 and 26) and predisone (1 mg/kg/day orally on days one to 14, with a decrease to one-third of the dosage on days 15–28) and acquired complete response after 55 days.

## Discussion

Several cases of hyperleukocytosis have previously been reported. A four-year-old male was diagnosed with ALL with hyperleukocytosis (WBC count of 85.8×10^9^/l). Brain CT scan showed multiple intracerebral haemorrhages, while chest CT showed a parenchymal right basal consolidation and diffuse infiltration. Following emergency treatment and combination chemotherapy, the male achieved complete remission (1). In an additional report (5), a 19-year-old male was diagnosed with ALL (WBC count of 121.0×10^9^/l). Brain CT showed multiple areas of parenchymal haemorrhage with intraventricular hemorrhage and subdural hematoma along the posterior falx. However, the male was declared brain dead and care was withdrawn.

In the present study, the two cases of acute lymphocytic leukemia were not complicated by leukostasis syndrome, possibly due to lower WBC counts. Of the seven cases of reported acute myeloid leykemia with hyperleukocytosis (WBC count of 104–339×10^9^/l), six were complicated by leukostasis syndrome. The clinical presentation included dyspnoea/lung failure, as well as confusion, dizziness, headache, tinnitus, blurred vision, somnolence, stupor, delirium, coma and ataxia. All seven patients were treated with leukocytapheresis. Further treatment was based on chemotherapy alone, and leukapheresis treatment was terminated to avoid interference with the chemotherapy. Two of the patients critically ill on admission to hospital succumbed during the first week following diagnosis of leukaemia, while five patients acquired complete remission (6). In the present study, the two patients with acute myeloid leukaemia complicated with leukostasis syndrome abandoned all treatment and were discharged from hospital. While case one succumbed at hosptital during the first week following the diagnosis of leukaemia. Therefore, leukostasis syndrome is an emergency case.

Hyperleukocytosis is defined as a WBC count of >100×10^9^/l (100,000/μl). The incidence ranges between 5 and 13% in adult AML, and between 10 and 30% in pediatric and adult ALL (7). This laboratory abnormality can cause severe mortality by inducing one or more of the following: Leukostasis, tumor lysis syndrome and disseminated intravascular coagulopathy (DIC) (8).

Hyperleukocytosis has significant prognostic implications with or without one of the clinical complications mentioned above. For prognostic assessment, the common arbitrary leukocyte count cut-off is 50,000/μl for AML and 400,000/μl for ALL. Myeloblasts are larger in size than lymphoblasts and lymphocytes; therefore, leukostasis is more frequent in AML than in ALL and CLL. Hence, if hyperleukocytosis occurs in lymphatic leukemias, it does so at markedly higher WBC counts (9).

The pathophysiology of leukostasis is not clear. There are two leading theories. The rheological theory relates to the principle that blood viscosity is a function of two factors, the deformability of individual cells and the volume of the cell fraction in the blood. Blasts are less deformable than mature WBCs. For elevated WBC counts, the high fractional volume of leukocytes (leukocrit) results in increased blood viscosity. As a result, the non-deformable blasts can occlude microvessels and reduce flow in the vessels with marginally larger caliber. The other theory is that endothelial cells activated by blasts secrete cytokines (in particular, TNF-α and IL-1β), and blast-endothelial cell interaction mediated by specific adhesion receptors (selectins and VCAM-1) play a significant role in promoting blast cell recruitment (10,11). Both mechanisms lead to vascular obstruction, which induces tissue hypoxia.

Leukostasis leads to vascular obstruction, and the central CNS and lungs are the most common sites for this (12). The CNS symptoms may include confusion, dizziness, headache, tinnitus, blurred vision, somnolence, stupor, delirium, coma and ataxia. On examination, focal deficits may be elicited and retinal hemorrhages may be present (13). CT scan or magnetic resonance imaging of the head may reveal intracranial hemorrhage. Respiratory symptoms include dyspnea, tachypnea and hypoxemia, with the presence of auscultatory rales. A chest X-ray or a CT scan often will show bilateral interstitial or alveolar infiltrates (3).

In AML, the majority of studies have found that hyperleukocytosis is a poor prognostic factor (13). AML patients with hyperleukocytosis have demonstrated lower complete remission rates, disease free survival and overall survival, as well as high rates of early mortality. In ALL, there are consistent data regarding the poor prognosis of hyperleukocytosis. It is unknown whether the prognostic effect of hyperleukocytosis is a result of high tumor burden or whether those leukemias with high WBC counts are distinct entities with different pathophysiology and clinical characteristics. It may be, for example, that hyperleukocytosis is an expression of a molecular change, such as the FLT3-ITD mutation in AML, and that the molecular aberration itself is responsible for the poor prognosis rather than the actual WBC count. In several leukemias, there is an association of hyperleukocytosis with specific subtypes of the disease. For example, several studies have linked hyperleukocytosis to monocytic differentiation subtypes, particularly M4Eo and M5a in AML, and M3v in acute promyelocytic leukemia, while in ALL there is an association with t(4:11) and t(9:22) (13).

The management of hyperleukocytosis includes intensive supportive care and cytoreduction. Supportive care consists of prevention of tumor lysis syndrome by aggressive hydration and allopurinol, and respiratory support as required. The cytoreduction may be achieved by leukapheresis, induction chemotherapy and hydroxyurea. A critical problem is that if the WBC counts are not reduced prior to induction therapy, tumor lysis syndrome and DIC may be aggravated with the induction treatment. In AML, leukapheresis and hydroxyurea are used to cytoreduction; while in ALL, leukapheresis and dexamethasone are used (13).

In the present case series, all five patients presented with hyperleukocytic leukemia. Cases 1, 2 and 3 were AML with induced leukostasis syndrome, while cases 4 and 5 were ALL without leukostasis syndrome. The reason is that myeloblasts are larger in size than lymphoblasts and lymphocytes. The sites of leukostasis were the lungs in case 1 and the CNS in cases 2 and 3, which were both invalid. Hence, physicians should begin aggressive treatment for leukostasis when the first respiratory or neurological symptoms or sign appear in a leukemic patient with hyperleukocytosis. In the present study, the three AML cases of hyperleukocytosis were of the M5 subtype and the two ALL cases of hyperleukocytosis were BCR/ABL fusion gene-positive. This suggested that the poor prognosis of hyperleukocytosis may be linked to the monocytic differentiation subtypes in AML, while in ALL there is an association with t(9:22). This is consistent with previous reports in the literature (3,12,13).

In conclusion, hyperleukocytosis has a significant prognostic implication. Leukostasis syndrome frequently arises in hyperleukocytosis, with common sites including the lung and CNS. Leukostasis syndrome is an emergency case with a high rate of early mortality, therefore, the management must be aggressive. The overall aim is to reduce early mortality, however, the pathophysiology of leukostasis remains unclear and future studies are required.
